# The use of predator tags to explain reversal movement patterns in Atlantic salmon smolts ( *Salmo salar* L.)

**DOI:** 10.1111/jfb.15658

**Published:** 2024-01-16

**Authors:** C. Waters, D. Cotter, R. O'Neill, A. Drumm, J. Cooney, N. Bond, G. Rogan, N. Ó' Maoiléidigh

**Affiliations:** ^1^ Marine Institute Furnace, Newport Co Mayo Ireland

**Keywords:** acoustic telemetry, Atlantic salmon, migration, predation, predator tag, smolt

## Abstract

Acoustic telemetry has seen a rapid increase in utility and sophistication in recent years and is now used extensively to assess the behavior and survival rates of many aquatic animals, including the Atlantic salmon. As part of the salmon's complex life cycle, salmon smolts are thought to make a unidirectional migration from fresh water to the sea, which is initiated by changes in their physiology. However, some tag movement patterns do not conform with this and can be difficult to explain, particularly if the tagged fish has been eaten by a predator. This study combines the use of predator tags with machine learning techniques to understand the fate of migrating salmon smolts and thereby improve estimates for migration success. Over 3 years between 2020 and 2022, 217 salmon smolts (including wild and hatchery‐reared ranched fish) were acoustically tagged and released into an embayment on the west coast of Ireland. Some tagged smolts were observed to return from the estuary back into a saline lagoon through which they had already migrated. To distinguish between the movement of a salmon smolt and that of a predator, predator tags were deployed in migrating smolts in 2021 and 2022. The addition of a temperature sensor in 2022 enabled the determination of predator type causing the returning movement. A significant number of predator tags were triggered, and the patterns of movement associated with these triggered tags were then used with two types of machine learning algorithms (hierarchical cluster analysis and random forest) to identify and validate the behavior of smolts tagged without extra sensors. Both models produced the same outputs, grouping smolts tagged with predator tags with smolts tagged without the additional sensors but showing similar movements. A mammalian predator was identified as the cause of most reversal movement, and hatchery‐reared ranched smolts were found to be more likely predated upon by this predator than wild smolts within the lake and the estuary. However, overall migration success estimates were similar for both wild and hatchery‐reared ranched fish. This study highlights the value of predator tags as an essential tool in the overall validation of detection data.

## 1 INTRODUCTION

Biotelemetry and biologging allow scientists to remotely monitor the behavior of individual animals throughout their natural environment (Cooke et al., 2012). Generally, telemetry requires the use of an electronic tag such as radio, acoustic, or satellite tag attached to the species of interest, and detection data collected provide information on movement (Hazen et al., 2012). Advances in telemetry technology over the past 60 years (Hockersmith & Beesman, 2012; Lennox et al., 2017) and recent technological innovations have now made it possible to successfully investigate fish ecology on a new level (Crossin et al., 2017; Hussey et al., 2015). As a result, the scope and scale of fish migration studies have become more ambitious and are delivering important information, particularly for highly sensitive species that cannot be studied using conventional sampling techniques (Block et al., 2019; Johnston et al., 2022; Wright et al., 2022).

One such species, the Atlantic salmon (*Salmo salar*, Linnaeus, 1758), an anadromous salmonid native to the North Atlantic, is of biological, economic, and cultural importance. Atlantic salmon populations have been in decline since the mid‐1980s, based on evidence from the return rates of adults (Chaput, 2012; NASCO, 2019). Initially the cause of this decline was associated with overfishing, pollution, and barriers such as dams that cannot be bypassed during migration (Forseth et al., 2017; Parrish et al., 1998). Despite efforts to mitigate against these factors, such as fishery restrictions and closures leading to substantially less catch, salmon stocks are still constrained at sea (ICES, 2021), and there has been no consistent improvement reported in adult return rates (NASCO, 2019). Recent studies have inferred that the reason for this decline may be linked to several additional causes, including climate change and availability of prey sources (Todd et al., 2021; Utne et al., 2022); salmon aquaculture (Ford & Myers, 2008); marine predation (StrØm et al., 2019); and Illegal, unreported, and unregulated fishing (Dadswell et al., 2022). As a result, information on salmon ecology and distribution in the marine environment may be essential for the effective conservation and management of Atlantic salmon (Rikardsen et al., 2021). Although little is known of the distribution of salmon within the marine environment, distribution has been inferred using genetic assignment of captures at sea and models based on oceanographic variables (Bradbury et al., 2021; Gilbey et al., 2021; Mork et al., 2012).

Many studies have highlighted the high level of mortality at the critical post‐smolt stage, as salmon migrate for the first time to the marine environment through estuarine and inshore waters (Crozier et al., 2018; Flávio et al., 2020; Halfyard et al., 2012; Thorstad et al., 2012). These mortalities have been associated with both natural and anthropogenic influences such as predation, parasites, competition, by‐catch, and aquaculture interactions (Crozier et al., 2018; Falkegård et al., 2023; Flávio et al., 2021; Hansen et al., 2003; Johnsen et al., 2021), which have a fundamental impact on the abundance of adult returns to fresh water (Thorstad et al., 2012). Understanding the causes of mortality can shed light on potential population pinch points, especially when migratory survival has known population‐level effects (Chavarie et al., 2022; Gilbey et al., 2021; Vøllestad et al., 2009).

The use of acoustic telemetry as a means to monitor the outward migration of salmon smolts from river to the marine environment has become increasingly valuable in examining migration timing, survival rates, and potential environmental drivers of movement (Chaput et al., 2019; Lilly et al., 2022; 2024; Lothian et al., 2018). The movement of salmon smolts from river to the marine environment is accepted as a unidirectional movement with little deviation from the ultimate goal of reaching the sea, although studies have shown that the negotiation of standing water bodies can hinder this direct progress (Honkanen et al., 2018; Lilly et al., 2021). The importance of interpreting detection data correctly and accounting for unusual behavior or mortality is essential to prevent biased results (Hanssen et al., 2022; Klinard & Matley, 2020). Failure to identify detections associated with predation, for example, can cause a predation bias (Daniels et al., 2019; Hanssen et al., 2022). Reversals in direction of movement or unusual prolonged timings in salmon smolt detections should be examined and a quality control process applied (Buchanan & Whitlock, 2022; Daniels et al., 2018; Gibson et al., 2015; Romine et al., 2014; Villegas‐Ríos et al., 2020). Due to advances in telemetry technologies, studies have started to take advantage of new “predator tags” (i.e., tags that can record when digestion of the tag takes place and alter the coded signal to indicate this) to support the assessment and validation of fish movement (Daniels et al., 2019; Notte et al., 2021). From laboratory trials (Lennox et al., 2021; Schultz et al., 2017) to experiments in the wild, these once‐novel tags (Weinz et al., 2020) have proven to be another effective tool in the acoustic transmitter toolbox.

In this study, we describe the salmon migration of both wild and hatchery‐reared (from now on referred to as ranched) smolts from a brackish lake and estuarine habitat to the marine environment and inshore coastal waters of Clew Bay in the west of Ireland. We assess the potential bottlenecks to survival and determine the effects of predation using acoustic tags with extra sensors for digestion and temperature (predator tags). We test the hypotheses that migrating smolts make a unidirectional movement pattern and that other unexpected movement patterns are likely to be a consequence of predation. We test the hypothesis that acoustically tagged smolts without extra sensors can also be identified as having been predated by using machine learning outlined by Notte et al. (2021) while also examining how incorporating wrongly identified detections could over inflate survival estimates. Finally, we determine whether fish origin (wild or ranched) has an impact on performance.

## 2 METHODS

### 2.1 Study area

**FIGURE 1:**
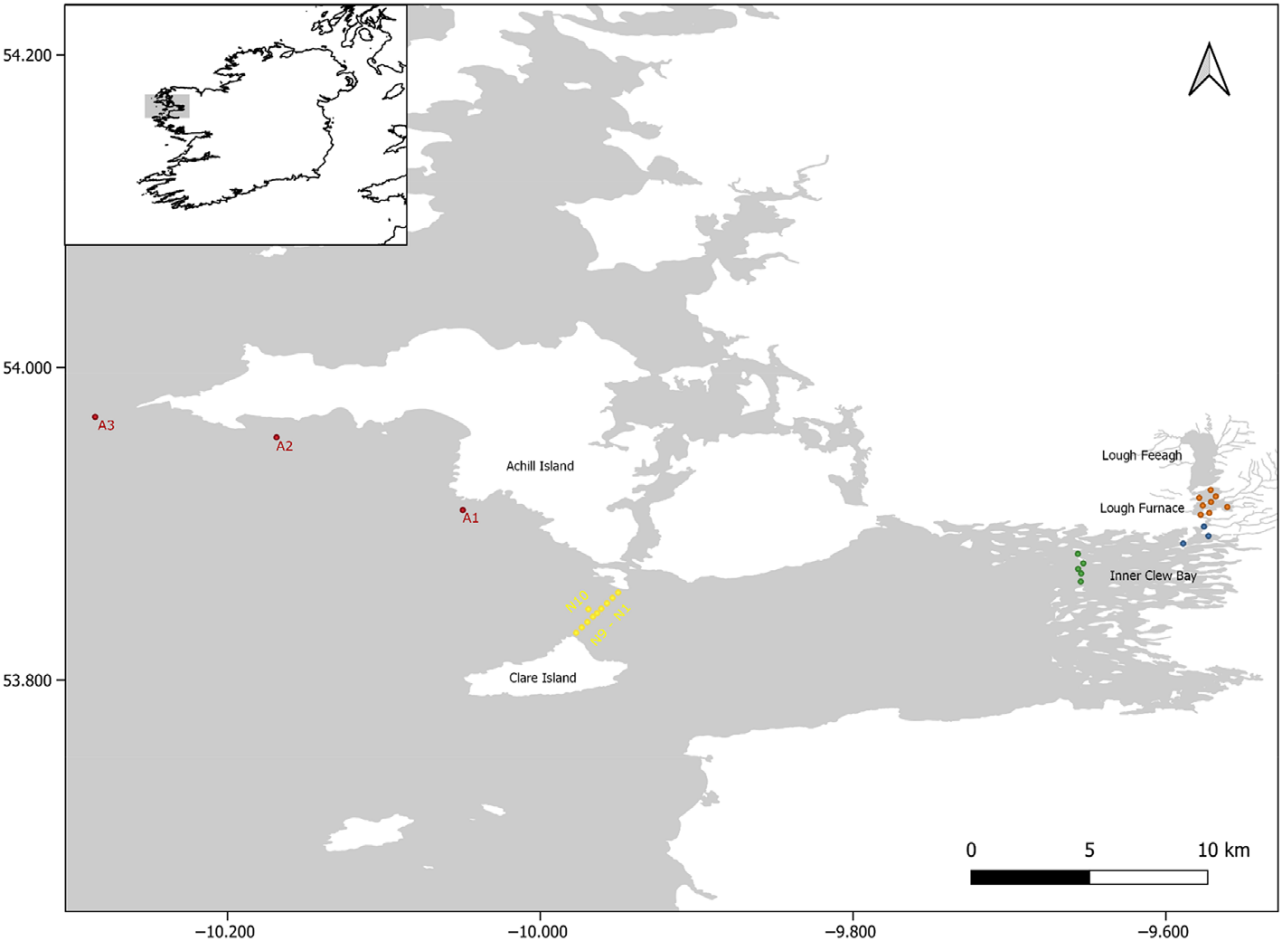
Map of the entire receiver array. Orange denotes lake receivers (F1‐8), blue is estuarine (E1 and E2, M1), green is Inner Clew Bay (M2‐6), yellow is Clare Island (N1‐10), and red is Achill (A1‐3).

**FIGURE 2:**
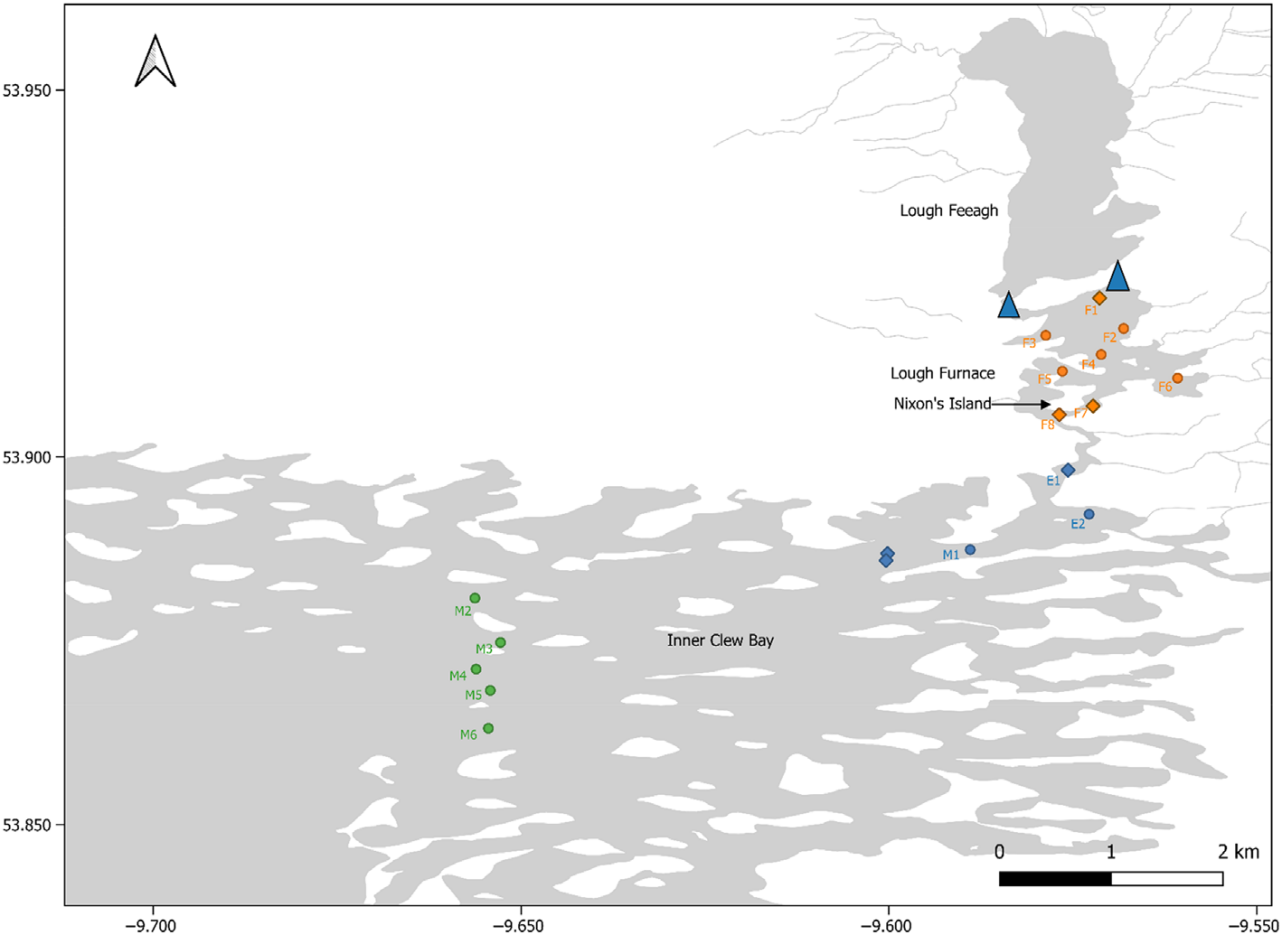
Close‐up of Lough Furnace, estuary, and Inner Bay receivers. Blue triangles represent location of traps. Receiver locations represented by a diamond indicate the six receivers also used in 2020. Orange receivers denote lake receivers, blue denotes estuary, and green denotes Inner Bay. The two diamond‐shaped blue receivers at the end of the estuary were not used in 2021 or 2022.

The study was conducted within the Burrishoole Catchment and Clew Bay located in County Mayo, Ireland (53^o^54′ N, 9^o^34′ W; Figures 1 and 2).

The Burrishoole Catchment is *c*. 100 km^2^ consisting of three main lakes, Bunaveela (46 ha), Feeagh (410 ha), and Furnace (a brackish coastal lagoon, 141 ha). The catchment is drained by 45 km of rivers and streams. It is an extensively grazed area of upland peat and coniferous plantation forestry. Lough Feeagh is oligotrophic and is 45 m at its deepest part. It drains into Lough Furnace via two outlets: the Salmon Leap and the Millrace, both of which have operational upstream and downstream Wolf‐type fish traps. Lough Furnace is a tidal saline lagoon (noted for its anoxic deep waters), fed by fresh water from Lough Feeagh and saline water from Clew Bay through the Burrishoole Estuary. The estuary is shallow (*c*. <2 m) and is 3.3 km in length connecting to Clew Bay, a westerly facing bay connecting out to the North Atlantic. Salt water transported into Lough Furnace is constricted by the topography of Nixon's Island before reaching the inner basin.

### 2.2 Salmon stock

Two specific groups of smolts were tagged in this study, that is, wild and ranched salmon. Wild salmon migrating predominantly as 2‐year‐olds were taken from downstream traps between Lough Feeagh and Furnace. In the 1960s a ranched breeding population was established from wild adult salmon returns to the Burrishoole Catchment and have been line‐bred ever since. Salmon ranching is defined as “the release of reared juvenile Atlantic salmon with the intention of harvesting all of them on their return” (Anon., 1994). Groups of 1‐year‐old ranched smolts are released into Lough Furnace every year, as part of the Irish National Coded Wire Tagging and Tag Recovery Programme, to coincide with the wild smolt run in April and May (Cotter et al., 2022).

### 2.3 Acoustic tagging

During the smolt migration run of 2020, 2021, and 2022, 217 salmon smolts were acoustically tagged (Table 1) and released as part of the SeaMonitor project based at the Marine Institute Catchment, Research Facility. Over the 3‐years, 162 ranched and 55 wild salmon smolts were tagged. Four types of Innovasea (Halifax, Nova Scotia, Canada) acoustic tags were used at 69‐kHz frequency: V7‐2x, V8‐4x, V7D‐2x, and V7DT‐2x (Table 1). Predator tags (V7D‐2x and V7DT‐2x) were used to assess the impact of predation on ranched salmon smolts. The predator tag is triggered when a polymer coating is digested (due to temperature and biological variables in the predator's gut). At this point the tag changes its digestive sensor value and records time since triggering, and the sensor value is then detected and recorded by the receivers when in range. Upon download of detection data, each detection of a predator tag will provide extra information on whether the tag is in process of being triggered or time passed since its triggering. The temperature sensor of these tags takes the ambient temperature of its surroundings and therefore may be able to inform as to predator species. In a fish this can depend on water temperature (Simpson, 1908) from this region at the time of the study, which may be between 10 and 15°C, whereas bird and mammal internal temperature is independent of their environment. Core temperature of birds can range between 34 and 44°C, and that of mammals can be from mid‐30°C to upper 30°C (Clarke & Rothery, 2008).

**TABLE 1:** Tag and salmon smolt information for all tagged smolts 2020–2022.

Year	Smolt origin	Number tagged	Mean fork length (mm)	Tag type	Output power (db)	Tag diameter (mm)	Tag interval (s)	Tag life (days)	Tag weight, g (in air)
2020	Ranched	37	196.70 ± 3.38	V7‐2x	137	7.0	20–40	69	1.5
2021	Ranched	50	195.84 ± 2.81	V8‐4x	144	8.0	40–80	173	2.0
	Wild	25	151.40 ± 3.95	V7‐2x	137	7.0	40–80	120	1.5
	Ranched	10	206.10 ± 12.04	V7D‐2x	137	7.0	30–90	100	1.7
2022	Ranched	50	213.70 ± 3.39	V8‐4x	144	8.0	20–40	98	2.0
	Wild	30	166 ± 4.34	V7‐2x	137	7.0	20–40	69	1.5
	Ranched	15	213.87 ± 5.71	V7DT‐2x	137	7.0	20–40	57	1.7

On the day of tagging, smolts were anaesthetized using 0.1 g/L MS‐222 buffered with 0.1 g/L of sodium bicarbonate. Stage three of anesthesia (loss of equilibrium, no reaction to touch stimuli) was reached within *c*. 5–6 min. Fork length (FL, mm) and weight (±0.1 g) were recorded for each individual. Only salmon smolts measuring ≥140 mm were selected for tagging. Once anaesthetized, the fish were placed ventral side up on a V‐shaped trough for stability. A tube was inserted into the fish's mouth, and a stream of diluted anesthetic was passed across its gills. An incision (1.5 times the diameter of the tag) was made on the ventral side of the abdominal wall into the intraperitoneal cavity, and the tag was inserted. The incision was then closed by two interrupted surgical knots using 4/0 vicryl sutures. Smolts were then placed in recovery tanks and assessed until normal swimming behavior had resumed. All ranched smolts were held overnight in flow‐through hatchery ponds and released the next day. Wild fish were assessed differently to minimize the time held in an artificial environment. If wild smolts were deemed to have recovered, they were released within 2 h of tagging; otherwise they were held overnight and reassessed the next day. Any mortalities were recorded and tags redistributed. Tagging was conducted under Health Products Regulatory Authority (HPRA) license number AE19121/P003, case number 7028960.

### 2.4 Receiver deployment

Innovasea receiver models VR2W and VR2Tx at 69‐kHz frequency were deployed in this study. Receivers were deployed after range testing was consistent with the findings outlined by Doogan et al. (2023), in which extensive range tests were conducted using V7‐2x tags in the same locality. Receivers on sections of each array were strategically situated no further than 400 m away from each other or 200 m from land mass to optimize the chance of detections.

In 2020, due to COVID restrictions, receiver deployment was reduced, and six VR2Ws were deployed in the lake and estuary only. When COVID restrictions were lifted, the receiver array was expanded to include additional lake receivers, the inner section of Clew Bay, Clare Island, and Achill (Figure 1). A total of 29 receivers were deployed in both 2021 and 2022; these included the same receiver locations as 2020 apart from 2 at the end of the estuary (see Figure 2).

In 2021 and 2022, lake, estuary, and Inner Bay receivers were deployed for 40–56 days and Clare Island receivers for 20–30 days. Receivers were not placed on the southern side of Clare Island; as in previous studies in 2017 and 2018 (Doogan et al., 2023), it was found that the majority of smolts (>91% in 2017 and 100% in 2018) used the northern pass of Clare Island, potentially following stronger outgoing currents (Nagy et al., 2023).

In 2021, after a storm event, two receivers at Clare Island came away from their moorings; one of these receivers was recovered, but the other was not.

### 2.5 Data analysis

All acoustic data were compiled using Innovasea proprietary VUE software. Before data were exported into csv format, a false detection analysis was run within VUE. Interference between transmitter signals or incomplete transmissions were confirmed as false or erroneous detections and were manually removed from further analysis. All analysis was conducted in R, version 4.2.0 (R Core Team, 2022), and visualized using the package “actel” (Flávio & Baktoft, 2021). Further visualization of data from predator tags was conducted using the “Refined Shortest Path” analysis and plotTracks function (Niella et al., 2020) and the Circular statistics package “circular” (Agostinelli & Lund, 2022).

### 2.6 Movement patterns

Two types of movement patterns are described, unidirectional and reversal/returning movement. Unidirectional movement describes salmon smolt movement in which on leaving the lake, smolts make a direct movement out of the estuary and through the other receiver arrays. Smolts may spend a limited time detected on a receiver array, but in general, unidirectional movement means they are actively migrating to sea. Reversal/returning movement describes any backward movement of smolts between receiver locations. For example, after leaving the lake and being detected on estuary receivers, tags are then detected again on lake receivers, or after leaving the estuary and detected on Inner Bay receivers, tags are detected again on estuary receivers.

### 2.7 Cluster analysis

Cluster analysis was run to assess whether any of the salmon smolts without extra predator sensors displayed the same movement patterns as those confirmed as being predated on using predator tags. All salmon smolts in 2020, 2021, and 2022 that were detected beyond lake receivers were used in this analysis (*n* = 175). As the receiver array was different in 2020, data for this year (*n* = 33) were run separately to data from 2021 and 2022 (*n* = 142).

Cluster analysis was chosen to group similar behaviors together as it is an unsupervised machine learning technique, meaning that there is no responsive variable for a given dataset. The three main steps of the cluster analysis were to (1) select appropriate variables to include, (2) select a dissimilarity matrix, and (3) select a method of clustering (per Gibson et al., 2015). Nine variables were chosen based on detection patterns and set in a data matrix. These included total number of detections, total time detected (h), velocity (maximum upstream speed, mean upstream speed, maximum downstream speed, and mean downstream speed) in meters per second, total number of reversals (change in direction), residency in lake (h), and residency in estuary (h). In 2021 and 2022, two more variables were added: time (h) in which it took tag to trigger from release (predator tags only) and residency within the Inner Bay (h). The migration function in the “actel” package was used to calculate residency within the sections of the array and speed between the last lake receiver and the first estuarine receiver.

Variables for which there were no data were assigned a zero (e.g., no upstream movement, as movement was downstream only). Data were then both centered and scaled, and the difference between variables was analysed using Euclidean distances (Gibson et al., 2015). A hierarchical clustering approach was adopted using the “factoextra” package (Kassambara & Mundt, 2020) and the “hclust” base R function, and Ward's principle of minimum variance was selected (Ward, 1963). The k‐means clustering score techniques, elbow method, and silhouette analysis were used to confirm the optimal number of clusters created by the hierarchical dendrogram. Finally, to test the sensitivity of the cluster analysis to the migration variables used, a number of trials were run in which varying combinations of variables were removed from the analysis. Assumptions of predation were made when tags without the extra predator sensors were grouped/clustered with those confirmed to have been triggered. These detections were then identified as no longer emitting from a live salmon. The key metrics that identified clusters as predators or smolts in 2021 and 2022 were visually compared to the clusters in 2020 data to confirm whether these clusters could be considered as a predator group or a smolt group, as no predator tags were used in 2020.

### 2.8 Random forest analysis

To determine whether cluster analysis was the best method of predicting the fate of salmon smolts not tagged with predator tags, random forest (RF) analysis, a type of supervised machine learning, was run using the same variables as the cluster analysis. Data from 2020 were not used in this analysis as no predator tags were deployed and the receiver array was different. Only data from 2021 and 2022 were run through the RF model.

The RF model uses multiple classification trees on a dataset to increase the accuracy of predictions (Cutler et al., 2012). Methods used in this paper were similar to those outlined by Notte et al. (2021), in which data where the fate is known is used initially to train the model before its application to data where the fate is unknown to predict fates. The classification model estimates the relationship between a categorical variable and its numeric predictors, essentially predicting the target variable (a smolt or a predator). A confusion matrix is used to identify the effectiveness of the said model by analysing the error rate. The model was optimized by testing the fluctuations between out‐of‐bag (OOB) error and class error rate, when these were found to stabilize the number of trees chosen for the model was determined. To further reduce the amount of overfitting of the model to the dataset, the number of variables tested at each node (mtry) was chosen based on minimizing the OOB error. Class weight was calculated as the total number of observations per class divided by the number of classes multiplied by the observations in each class. Changing class weight resulted in no change in error values and was therefore not used in the final model. The parameters chosen for the optimal RF model are presented in Table 2. Model accuracy was tested using the area under the receiver operating characteristic curve and F1 score. Variable importance was determined by the mean decrease in accuracy and the mean decrease in Gini (decrease in node purity if the variable is not used).

**TABLE 2:** Random forest model metrics.

Parameter	2021 and 2022
ntree	50
mtry	5
Classwt c(Pred, Smolt)	NA
OOB error	1.3%
Class error c(Pred, Smolt)	0.08, 0

Abbreviations: Class error, class error for predators and smolts; Classwt, class weights assigned to predators or smolts; mtry, number of variables considered at each node; NA, not applicable; ntree, number of decision trees; OOB error, out‐of‐bag error rate.

### 2.9 Estimation of migration success

A successful migrating salmon smolt was initially defined by the presence of detections on a receiver or array. For instance, if it was detected at the last lake receiver (F8, lake exit), it was determined that it had successfully reached this location. If it was detected on one of the five Inner Bay receivers (M2–M6, Figure 2), then it was determined to have successfully reached that location and so on. The proportion of salmon smolts reaching that location was then calculated for each receiver or array. This was called the “biased” group. These figures were then reassessed once the machine learning had been executed. Machine learning included only smolts that were detected beyond lake receivers (*n* = 175) and grouped tags into either smolt or predator fates. This was called the “unbiased” group.

To account for detection efficiency of acoustic receivers (*p*) and migration success (Phi) through the array, a mark‐recapture model was used. A Cormack–Jolly–Seber model for live recaptures (Cormack, 1964; Jolly, 1965; Seber, 1965) adopted by other Atlantic salmon telemetry studies (Gibson et al., 2015; Larocque et al., 2020; Lilly et al., 2022) was applied. The CJS models (logit link) were fitted using maximum‐likelihood estimation for each year of the study and were run using the RMark package (Laake, 2013) in R, which is based on the MARK programme (White & Burnham, 1999). Receiver efficiency was determined as the percentage of smolts to be detected at receiver locations. Receiver locations used in this analysis were lake receivers (F1–F7), the last lake receiver (F8), estuarine receivers (E1 and E2), the last estuary receiver (M1), Inner Bay receivers (M2–M6), and Clare Island (N1–N10) (see Figures 1 and 2). In 2020, the receiver locations used only went as far as the last estuary receivers (Figure 2). In 2021 and 2022, receiver efficiency could not be determined for Clare Island receivers as there were no receiver lines beyond this point. Additionally, detection efficiency (*p*) was tested only against receiver locations. It is also worth noting that Inner Bay receivers (M2–M6) were not a full gate and did not span the entire bay.

Capture histories were created to evaluate migration success (as described by Larocque et al., 2020), and additional covariates were added to measure this success included: receiver location, fish origin (wild or ranched), FL, and tag to body mass ratio (TMR). Before model selection, goodness of fit of the global model (*ĉ*) was tested using a bootstrapping method (*n* = 1000 simulations) to calculate over‐dispersion, as discussed in Larocque et al. (2020) and Lilly et al. (2022). Models were run separately for each year of the study, and if quasi‐likelihood over‐dispersion parameter was greater than one (>1.00), the over‐dispersion parameters were adjusted to quasi‐likelihood AIC (Halfyard et al., 2013). Models were ordered based on quasi‐likelihood AIC (QAIC) values, and the lowest‐ranking model was chosen (based on a low QAIC value and highest model weight). Models were run for both biased and unbiased salmon detections.

Migration timing of successful salmon smolts (wild and ranched) was assessed between sections of the array, for example, from release to the last lake receiver, the last lake receiver to the last estuary receiver, the last estuary receiver to the Inner Bay receiver line, and so on. All section movement calculations were determined using the “actel” package in R. A two‐way ANOVA was run on dependent variable migration time (h) and independent variables year and origin (wild or ranched).

## 3 RESULTS

### 3.1 Movement patterns

**FIGURE 3:**
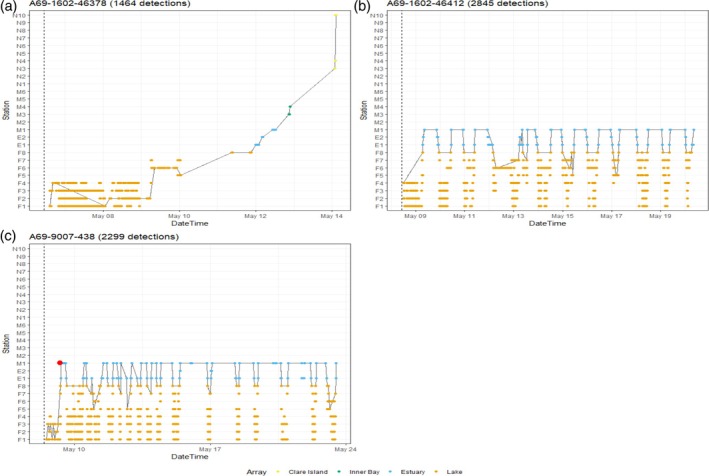
Example of three individual detection plots displaying movement patterns in a salmon smolts in 2021. (a) Unidirectional movement, (b) reversal movement, and (c) triggered predator tag movement (red dot represents time of tag triggering). The vertical dashed line indicates time of release; all dots represent a detection on a corresponding receiver station, and the solid line represents valid movement events as predicted by “actel”.

Both unidirectional and reversal patterns were observed in smolts tagged with and without predator sensors. In 2020, the movement patterns of 23 of the 37 ranched smolts displayed a unidirectional progression through the receiver array. However, movement patterns for seven ranched smolts displayed a “returning behavior,” meaning that once they had moved from the lake into the estuary, they made forays back in and out of the lake. In 2021, 22 of 85 fish displayed unidirectional movement as far as Clare Island (e.g., Figure 3a) and 18 ranched fish were identified as displaying the “returning behavior” (e.g., Figure 3b). In 2022, 49 of 95 fish displayed unidirectional movement as far as Clare Island. Eight ranched fish and one wild fish displayed “returning behavior”; this was the first of the wild cohort to have displayed this behavior in the 3‐year study. The remainder of the smolts in each study year were never detected as far as Clare Island receivers.

In 2021, 5 of the 10 ranched smolts tagged with predator tags were detected as triggered (e.g., Figure 3c). From a visual inspection of detection plots, the movement patterns of the returning smolts without predator sensors and those with triggered predator tag were similar. This pattern of behavior was also recorded in 2022, when 7 of the 15 predator tags were also triggered.

### 3.2 Behavior of smolts tagged with predator tags

In 2021 and 2022, 25 predator tags were used to assess predation. Nine of the 12 tags triggered were detected as eaten by a predator within the lake and 3 within the estuary (Table 3). In 2022, predator tags (*n* = 15) incorporated a temperature sensor, with all seven triggered tags recording a temperature >36°C, suggesting that the salmon smolts had been ingested by a mammal. Movement patterns of all triggered predator tags, apart from one in 2022, displayed the “returning behavior” visualized using actel detection plots. No triggered predator tags were detected leaving Clew Bay, and the farthest detection was at M6, an Inner Bay receiver.

**TABLE 3:** Summary data for triggered predator tags.

Type of tag	Location tag was triggered	Time between first detection and tag triggered (h)	Time of day detected as triggered	Average temperature before triggering (^o^C)	Average temperature after triggering (^o^C)	Total time recorded on array (days)
V7D‐2x	Lake	45.07	07:44:54	NA	NA	25
	Estuary	18.12	06:16:58	NA	NA	14.18
	Lake	61.95	01:53:02	NA	NA	10.33
	Estuary	53.55	17:04:43*	NA	NA	10.7
	Lake	12.33	23:19:59*	NA	NA	45.26
V7DT‐2x	Lake	16.13	02:43:43	12.26	36.52	32.28
	Lake	16.52	02:57:55	12.26	36.33	25.94
	Estuary	20.22	07:12:05	13.15	36.63	0.7
	Lake	17.40	02:30:17	12.41	36.74	2
	Lake	18.95	05:41:12	12.71	36.62	32.13
	Lake	12.63	23:14:28	12.88	36.53	2.14
	Lake	10.23	21:32:29	12.55	36.85	31.68

*Note*: Time detected as triggered indicates time at triggering, apart from two cases* in which the time it was first detected was >4 h since triggering, based on the sensor values.

Triggered predator tags were detected on all lake and estuary receivers at all times of the day and night (Figures S1a and S1b), and the tags displayed extensive movement between lake, estuary (Figures S1c), and even Inner Bay receivers.

During the analysis it was noted that data outputs from some triggered predator tags were similar, including date–time stamps at detection (Figures S2a–S2c). This infers that some of the triggered tags were likely to have been consumed by the same individual predator.

### 3.3 Machine learning

#### 3.3.1 Cluster analysis

**FIGURE 4:**
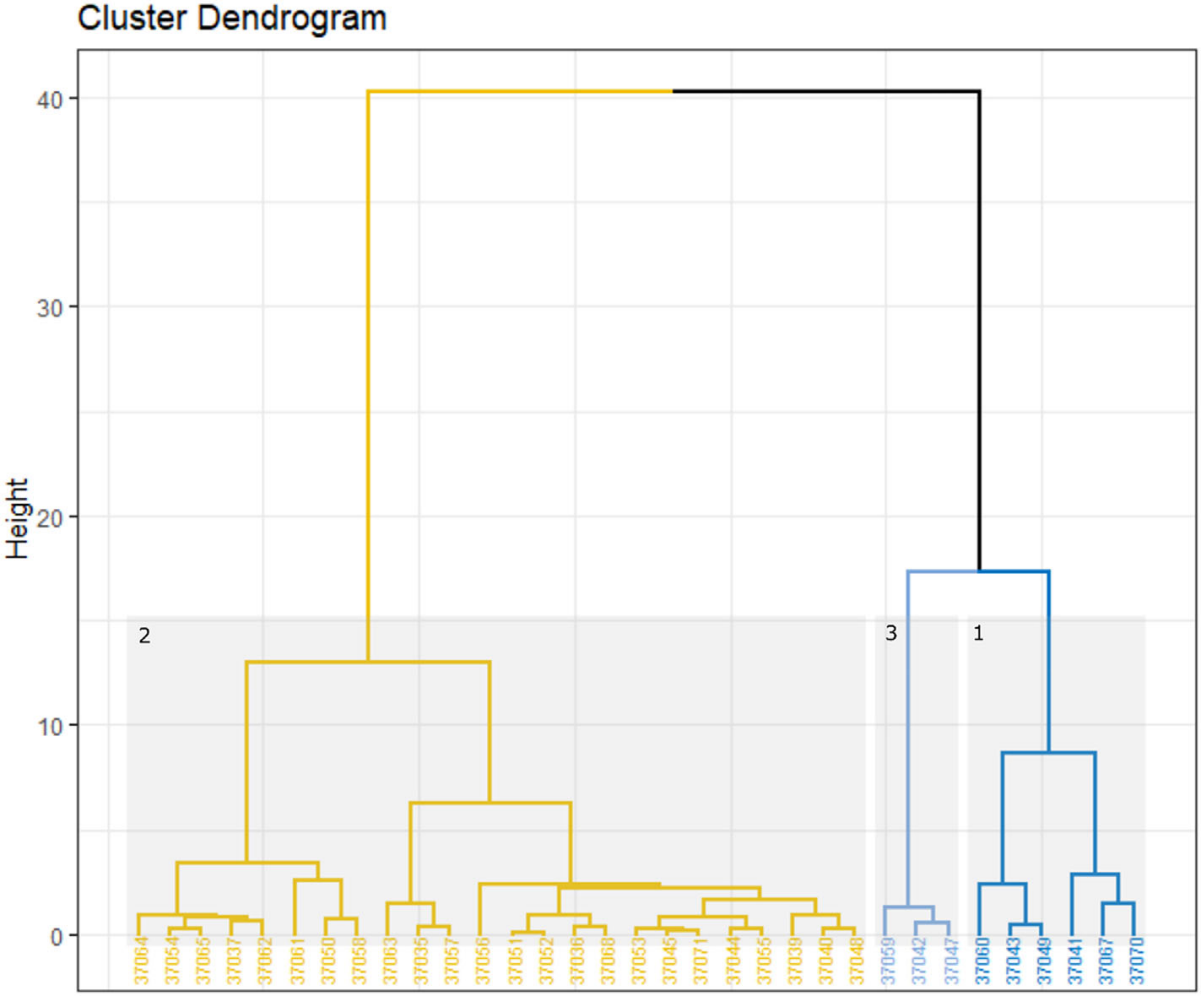
Hierarchical cluster dendrogram of 2020 transmitters. Yellow represents salmon smolts (cluster 2), and blue represents fish predated upon (clusters 1 and 3).

**FIGURE 5:**
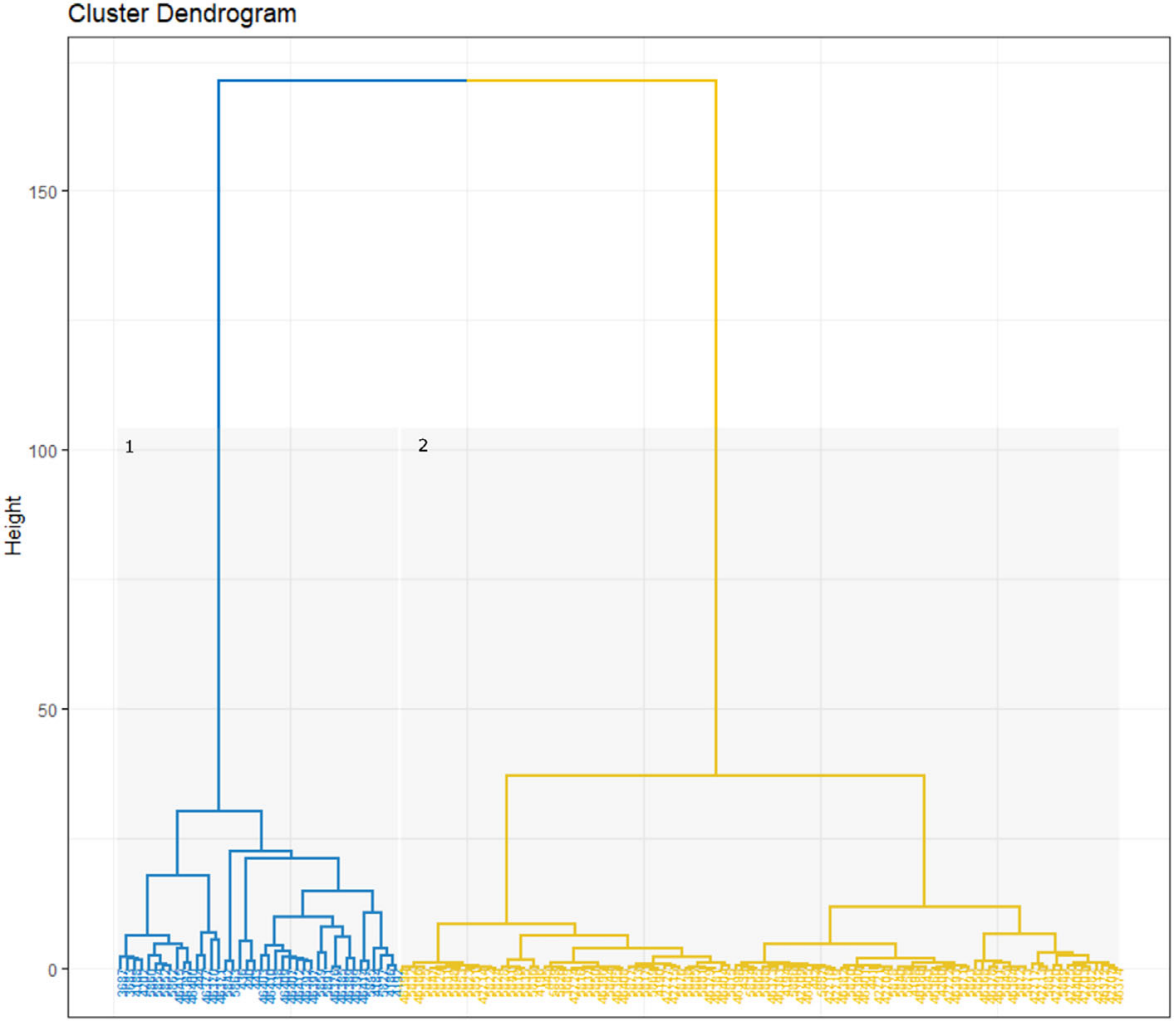
Hierarchical cluster dendrogram of 2021 and 2022 transmitters. Blue highlights fish predated upon (cluster 1), and yellow highlights salmon smolts (cluster 2).

The hierarchical cluster analysis created three major clusters for 2020 (Figure 4) and two for 2021 and 2022 (Figure 5). Cluster 1 represents salmon smolts that were confirmed as predated and those making the same returning movement patterns, and cluster 2 represents salmon smolts making a unidirectional movement. Cluster 3 in 2020 included fish (*n* = 3) that spent a prolonged period in the lake (Table 4) and could not be considered salmon smolts. These were likely to have been predated potentially by a different predator to those identified by cluster 1. Although predator tags were not used in 2020, comparison of the key metrics used to identify predators and smolts in 2021 and 2022 helped to distinguish the clusters produced by the analysis.

**TABLE 4:** Median and range (in parentheses) of migration metrics for each cluster identified by hierarchical analysis using 2020 data.

Migration metrics	Cluster 1 (*n* = 6)	Cluster 2 (*n* = 24)	Cluster 3 (*n* = 3)
Total number of detections	413 (224–617)	304 (127–992)	978 (913–1043)
Total time detected (h)	122.12 (112.38–189.37)	47.10 (19.06–129.01)	277.33 (274.52–277.68)
Mean downstream speed (ms^−1^)	1.36 (0.89–1.48)	0.58 (0^a^–1.1)	1.22 (1.14–1.23)
Maximum downstream speed (ms^−1^)	1.53 (0.89–1.61)	0.59 (0^a^–1.1)	1.22 (1.14–1.23)
Mean upstream speed (ms^−1^)	1.11 (0.74–1.45)	0 (0–0.21)	0
Maximum upstream speed (ms^−1^)	1.33 (0.94–1.45)	0 (0–0.21)	0
Total number of reversals	4 (2–8)	0 (0–2)	0
Time in lake (h)	93.5 (64.05–117.88)	45.59 (12.5–127.73)	254.30 (254.00–254.37)
Time in estuary (h)	13.87 (9.77–113.90)	1.82 (1.25–7.47)	23.32 (20.23–23.33)

^a^ Not detected at last lake receiver, therefore no speed calculated.

Clusters 1 and 3 were associated with a greater number of total detections and a longer time period detected on the arrays compared to cluster 2. In addition, in cluster 1 time periods spent within the lake and estuary and faster swimming speeds in both downstream and upstream movements were observed (Tables 4 and 5). Sensitivity analysis revealed that although each migratory variable chosen improved the robustness of the analysis, the speed variables had the most impact on the clustering of individuals. When the four speed variables were removed, 16 groupings occurred that did not fit in with the hypothesis that they were likely to have been predated. When only upstream speed metrics were removed, seven changes were identified. In all other trial scenarios, the clustering remained consistent. The cluster analysis also identified a predator tag in 2022 that had triggered but based on movement patterns would have been identified as a movement behavior by a salmon smolt due to its unidirectional movement. However, sensitivity analysis showed that by leaving in the variable “time tag was triggered” this individual was clustered in cluster 1, that is, confirmed as predated.

**TABLE 5:** Median and range (in parentheses) of migration metrics for each cluster identified by hierarchical analysis using 2021 and 2022 data.

Migration metrics	Cluster 1 (*n* = 40)	Cluster 2 (*n* = 102)
Total number of detections	2453.50 (181–30,446)	897 (133–5856)
Total time detected (h)	549.55 (37.09–1156.61)	71.36 (12.68–1068.06)
Mean downstream speed (ms^−1^)	1.16 (0.38–1.78)	0.49 (0–1.37)
Maximum downstream speed (ms^−1^)	1.71 (0.38–2.27)	0.51 (0–1.37)
Mean upstream speed (ms^−1^)	0.81 (0–1.63)	0 (0–0.3)
Maximum upstream speed (ms^−1^)	1.37 (0–2.1)	0 (0–0.3)
Total number of reversals	17.5 (0–51)	0 (0–2)
Time tag triggered (h)	17.76 (10.23–61.95)	0
Time in lake (h)	92.86 (11.67–737.19)	39.23 (1.52–154.4)
Time in estuary (h)	168.33 (1.63–1072.05)	3.26 (0.42–141.58)
Time in Inner Bay (h)	0 (0–620.08)	0.28 (0–32.6)

In all years, a small number of individuals (one ranched, two wild), despite showing reversals in movement pattern, were not clustered with the predator grouping (cluster 1), showing that smolts could also make reversals. However, no more than two reversals were identified as salmon smolts and no further upstream than the first estuarine receiver and the last lake receiver downstream (*c*. a 1‐km distance).

#### 3.3.2 Random forest

**FIGURE 6:**
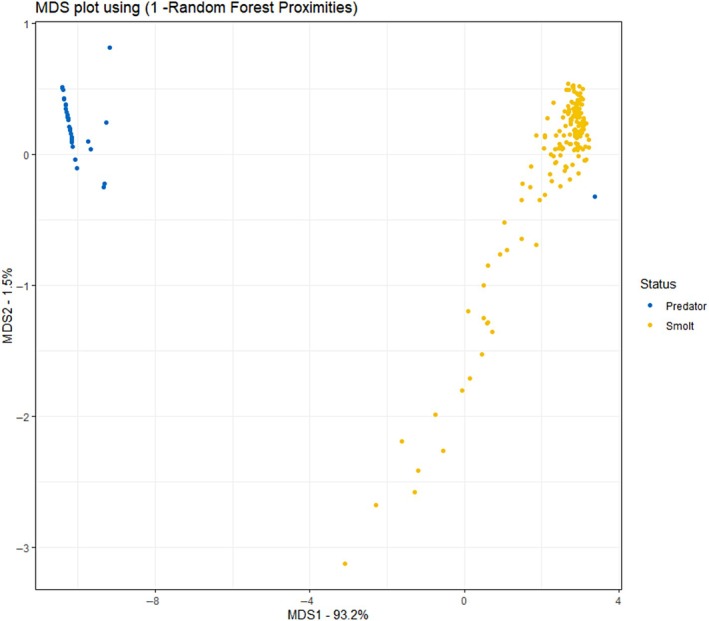
Multidimensional scaling (MDS) of random forest results of 2021 and 2022 transmitters. MDS is used to visualize information on the pair‐wise distances among individuals. The plot shows the similarity and dissimilarity between groups.

The model produced low class error and a predicted accuracy of 98.7% from the training set (Figure 6); changing class weight resulted in no change in error, and model accuracy tests indicated high model discrimination performance. The most important variables to classification were maximum downstream velocity, total number of reversals, and time spent in the estuary. Time tag was triggered was also an important variable but only for predator tags; it had no influence on the other tags without sensors.

The model classified all unknown tags (tags without extra sensors) as either smolt or predator according to the trained algorithm. Of the 2021 and 2022 data that were processed by this model, 100% of observations were classified into the same categories as the cluster analysis.

#### 3.3.3 Migration success

**FIGURE 7:**
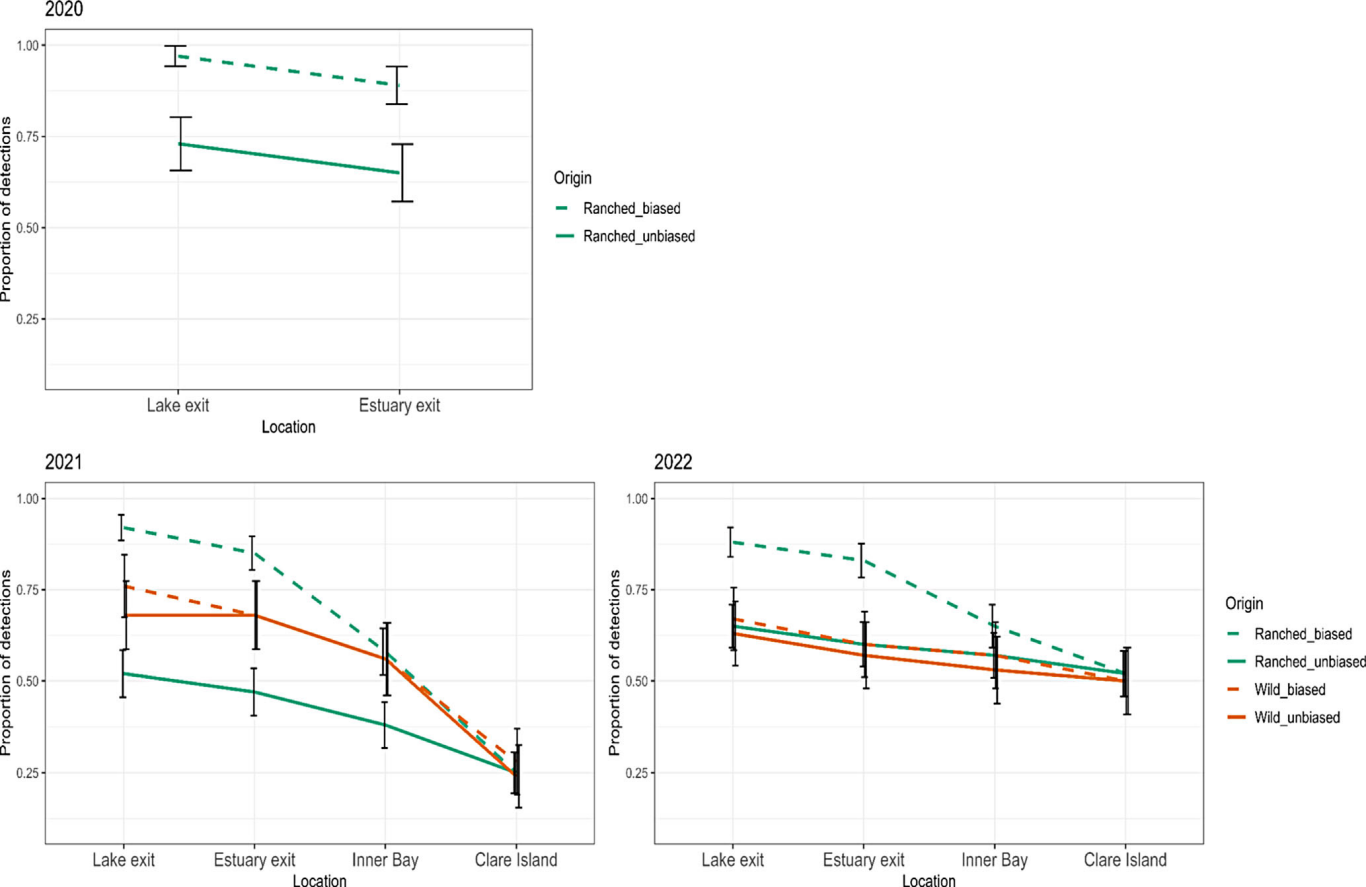
The proportion of tag detections for 2020–2022, at the lake exit, estuary exit, Inner Bay, and Clare Island. Line graphs show the unbiased results, incorporating predation into analysis (solid lines) or biased results, not including predation (dashed lines).

Over the 3‐years of study, one salmon smolt was undetected by receivers, and three fish were detected on only one receiver. These fish were assumed to be most likely tagging mortalities or predated upon by another type of predator. The number of smolts detected at receivers or receiver arrays was compared (Figure 7) between the predator‐biased group and the unbiased group. By incorporating the results of the machine learning analysis, the unbiased group indicates the closest estimate of survival at each location. It is evident that the ranched cohort was less successful at migration from the lake and estuary than the wild fish in 2021, with less than half migrating from the estuary (Figure 7). However, in 2022 the proportion of wild and ranched smolts migrating from those locations was comparable. In both 2021 and 2022, the proportion of wild and ranched salmon smolts detected at the final array, exiting Clew Bay (at Clare Island), was found to be similar (Figure 7).

The goodness of fit of CJS models found that in all years of the study lack of fit occurred, and therefore, QAIC was used instead of AIC model selection. The best‐fitting models for each year found that migration success from the lake to Clare Island was not dependent on FL, TMR, or origin of the salmon smolts. In 2020, the most parsimonious model for unbiased salmon detections suggested that migration success and detection probability were constant between receiver locations (Table 6). Model‐averaged migration success of salmon smolts between receiver locations was estimated to be 81% (c.i.: 65%–91%) and average detection probability as 100%. Biased results in 2020 averaged migration success as 99% (c.i.: 99%–100%). In 2021, the top candidate model for unbiased salmon detections suggested that migration success and detection probability varied across receiver locations. Model‐averaged migration success estimates ranged from 51% to 98% and detection probability from 86% to 100% across the receiver locations. Biased migration success results in 2021 averaged migration success ranged from 83% to 99%. In 2022, the best‐fitting model for unbiased salmon detections suggested that migration success was varied across receiver location and that detection probability was constant. Model‐averaged migration success estimates ranged from 64% to 96%, and detection probability was 99% (c.i.: 96%–100%). Biased results in 2022 averaged migration success ranged from 51% to 95%.

**TABLE 6:** Top five pool of Cormack–Jolly–Seber models for migration success and detection probability of salmon smolts acoustically tagged in 2020, 2021, and 2022.

Year	Model	Parameters (*n*)	QAICc	ΔQAICc	Weight
2020	Phi(.)*p*(.)	2	46.59	0	0.22
	Phi(Location)*p*(.)	4	47.16	0.58	0.16
	Phi(TMR)*p*(.)	3	47.45	0.86	0.14
	Phi(.)*p*(Location)	4	47.63	1.04	0.13
	Phi(FL)*p*(.)	3	47.81	1.23	0.12
2021	Phi(Location)*p*(Location)	10	276.83	0	0.73
	Phi(Location *Origin)*p*(Location)	15	280.16	3.33	0.14
	Phi(Location)*p*(.)	6	280.74	3.91	0.11
	Phi(Location * Origin)*p*(.)	11	284.15	7.32	0.02
	Phi(FL)*p*(Location)	7	305.21	28.38	4.42E‐07
2022	Phi(Location)*p*(.)	6	115.26	0	0.91
	Phi(Location)*p*(Location)	10	121.99	6.73	0.07
	Phi(Location * Origin)*p*(.)	11	123.95	8.71	0.03
	Phi(.)*p*(.)	2	129.19	13.92	5.97E‐10
	Phi(Location *Origin)*p*(Location)	15	130.70	15.44	0.002

*Note*: Models estimate migration success (Phi) and detection probability (*p*); covariates included receiver locations (Location), origin (wild or ranched) and their interaction, fork length (FL), and tag to body mass ration (TMR). Models were ordered based on quasi‐likelihood AIC (QAIC).

**FIGURE 8:**
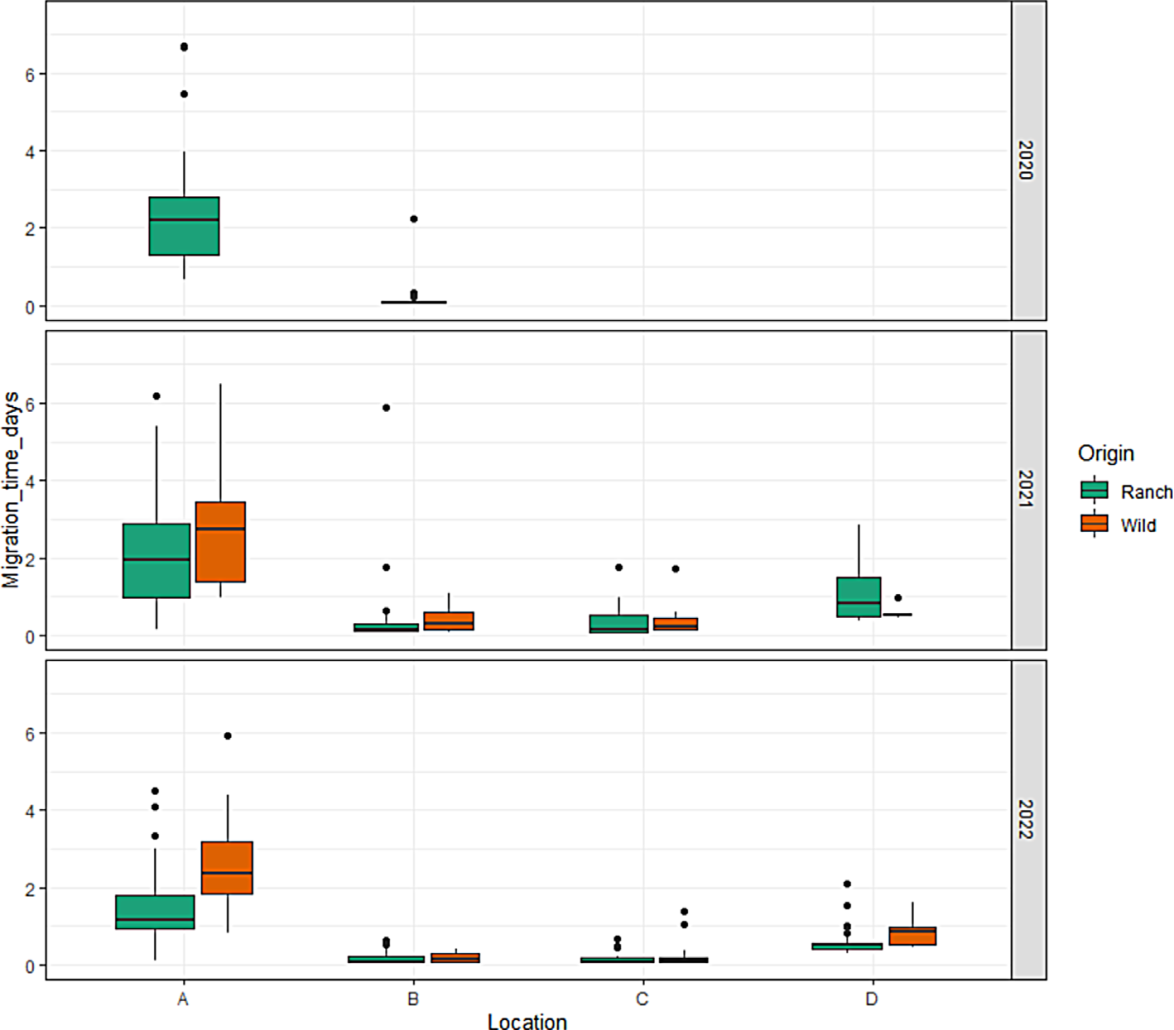
Migration time (in days) of wild and ranched salmon smolts between sections of array for 2020–2022. (A) From release to lake exit, (B) from lake exit to estuary exit, (C) from estuary exit to last Inner Bay detection, and (D) last Inner Bay detection to first Clare Island detection.

The migration timing of salmon smolts was examined for each section of the array over each year (Figure 8). Significant differences were observed in the time between release and lake exit between the tagged wild and ranched smolts (two‐way ANOVA: *F*(1) = 12.15, *p* < 0.001), with wild fish taking longer to leave the lake than their ranched counterparts. Migration time for both wild and ranched fish between the estuary and detection at the inner bay and detection at Clare Island were found to be significantly different between years (two‐way ANOVA: *F*(1) = 6.58, *p* < 0.01 [estuary to Inner Bay] and *F*(1) = 5.46, *p* < 0.05 [Inner Bay to Clare Island]).

## 4 DISCUSSION

### 4.1 Movement patterns and predator tags

The addition of predator tags to this salmon smolt migration study enabled the explanation of reversal movement patterns. This technology allowed us to show that most reversal movement patterns were associated with predation. Including predator sensors with temperature added another layer to results and enhanced potential identification of the predator species. In addition, these tags allowed a differentiation in movement patterns to be made between salmon smolts and a mammalian predator and enhanced overall survival estimates of salmon smolts in inshore coastal waters.

In each year migration timings showed that both wild and ranched Burrishoole smolts spent two days on average within the lake before onward migration and the majority of predator tags were triggered within the lake. Lake and estuarine habitats have previously been described as hazardous for salmon smolt migration (Honkanen et al., 2018; Kennedy et al., 2018). Handeland et al. (1996) suggest that a period of *c*. 48 h is needed for post‐smolts to become accustomed to saline conditions and that initial exposure to salt water can reduce antipredator avoidance with an increase in overall mortality. Acoustic telemetry has enhanced our understanding of smolt migratory behavior within lakes (Lilly et al., 2021), and using predator tags in this environment has shown how susceptible salmon smolts can be to predation within them (Hanssen et al., 2022).

Over the 3‐year study, 217 salmon smolts were tagged and released; however, only 175 were successfully detected on one or more estuarine receivers beyond the lake exit. It was determined that the most likely sources of mortality in the lake environment were either tagging mortality or predation. The returning movement patterns were predominantly displayed by the ranched cohort, with only one wild smolt identified as predated upon by the same predator. Avian, piscivorous fish, and mammalian predation is well documented in estuarine and marine areas (Dieperink et al., 2002; Hedger et al., 2011; Middlemas et al., 2003; Wheeler & Gardner, 1974). Wild salmon smolts have evolved to cope with such pressures, migrating in the hours of darkness, moving in shoals, and using the faster‐flowing upper water column (Lacroix et al., 2005; Moore et al., 1995; Thorstad et al., 2012). Ranched fish are potentially more vulnerable to predation than their wild counterparts, as they encounter predators for the first time (Einum & Fleming, 2001; Jackson & Brown, 2011). Predator training has been shown to improve this antipredator behavior in hatchery‐reared smolts (Jaervi & Uglem, 1993). The larger size of ranched fish has also been suggested to counteract the potential vulnerability of these post‐smolts (Ward & Hvidsten, 2011), but this could also make them more appealing to predators given the smaller size of the wild fish at this point of their migration.

In this study a predator tag with a temperature sensor was added to the project setup in 2022. The addition of this sensor showed that all returning movements were associated with a mammalian predator, and the frequent number of detections indicated that the harbor seal (*Phoca vitulina*) was the most likely predator, as Clew Bay has a known colony (NPWS, 2011). Normal body temperature for harbor seals is 36.5–37.9°C (van Wijngaarden et al., 2021), and all the temperature sensor values for our predator tags were >36°C.

Trials have proven that pinnipeds such as the gray (*Halichoerus grypus*) and harbor seals and California sealion (*Zalophus californianus*) can detect the acoustic frequencies of tags (at 69 kHz) within fish and use this information to prey more readily on tagged individuals (Cunningham et al., 2014; Stansbury et al., 2015). During this study, it became evident that tags could potentially be transmitting from the same predator and that a predator had several tags within its digestive tract. Seal predation on salmonids is commonly reported (Bendall & Moore, 2008; Berejikian et al., 2016; Flávio et al., 2020; Graham et al., 2011); however, whether these predators were targeting individual tagged smolts in this study is uncertain. Additional studies are required to determine whether the use of acoustic tags (at 69 kHz) might inflate the number of smolts predated upon by seals in the area.

Seal diet is known to have a seasonal variation (Hall et al., 1998), and the targeting of migratory species such as trout and salmon during their early marine migration has been reported (Mäntyniemi et al., 2012). Interestingly, this study showed that seal predation was concentrated within the lake and estuary. The detection of this behavior was in some cases prolonged, and the detection of seals was still ongoing 10–45 days after the digestion of tags. This would suggest a high level of usage by these predators within the inshore environment throughout smolt migration. Unlike Yurk and Trites (2000) who found harbor seals formed a feeding line across a river to predate on large numbers of salmon smolts, tracking seal movement in Clew Bay showed that the seals did not wait in one locality and wait for smolts to pass their location but showed a hunting behavior, utilizing all areas of the lake and estuary.

Seals are a natural predator of salmonids, and although it is evident that they are very adept at hunting salmon smolts on an annual basis, the geography of the Burrishoole locality provides a pinch‐point at which the migration route for the smolt becomes constricted between the lake and estuary. This area is influenced by the tide rather than currents that may make it easier for such predators to hunt.

### 4.2 Machine learning to validate detections

Predator tags were clearly efficient at identifying individual smolts that had been predated. However, further analytical steps were required to identify individuals without extra sensors that had been predated to derive accurate survival rates.

Daniels et al. (2019) and Notte et al. (2021) have reported on predator tags and machine learning as a means to validate salmon smolt movement. However, both these studies were based on striped bass (*Morone saxitilis*) as the main predator in Nova Scotia, Canada. Daniels et al. (2019) found that by using the predator tags, a predator‐bias in detection data could be identified. However, this bias was highest in the upper estuary and gradually declined over distance, with the short tag retention by predators associated with this decline in predator detections. Notte et al. (2021) recommend the use of machine learning to group fates of salmon smolts and had a greater success combining the predator tag data with a supervised RF model than the unsupervised k‐means cluster analysis. Although this study agrees with the use of machine learning to group fates, no difference was found between overall results produced in cluster and RF analyses. Predator tags can help train a supervised machine learning model, but error is still possible, as shown by a somewhat low 1.3% error rate in this study, created by a triggered predator tag that did not display the returning behavior identified in other tags. Only one variable (time tag was triggered) orientated this tag into the predator category, and removing this variable caused a misclassification in results. It is also important to note that reversals were identified in salmon smolt detections (nonpredated tags) but never more than two from the estuary to the lake and back again.

If there is a considerable difference in movement variables between species, as there is in this study, then a high degree of classification accuracy on training data should be expected (Daniels et al., 2019). The most important part of applying machine learning to detection data is the choice of input variables. The model needs to be able to identify a difference between unidirectional smolt movement behavior and something unexpected, such as the reversals. Choice of variables was based around those chosen by Gibson et al. (2015), but as this study was dealing with a different type of predator, some adaption was applied. A common important variable between the two models was velocity. Both upstream and downstream velocities were important to the classification of smolt fate, and when removed from the analysis, results were skewed. Upstream velocity was expected to be an important variable as predators made many more reversals (4–51) than smolts, which are generally expected to transit these locations rapidly (Thorstad et al., 2012). The migration metrics produced by the cluster analysis and describing the characteristics of each variable for either predators or smolts may contain a small number of measurements of the other target species or cluster. For instance, the metrics for a salmon smolt without the predator tags but classified as predated upon will include metrics from before it was eaten. Some salmon smolts classified as fish may also have metrics confounded by other types of mortality, as not all fish survived to Clare Island and as such will have shortened or censored detection histories. Therefore, a variable such as the mean downstream velocity should not be used to characterize predator behavior, and time on the array would not be helpful in interpreting smolt behavior. However, they are helpful for distinguishing between smolts and predators.

Variables included in machine learning are easily acquired when using acoustic transmitters, and using R packages such as actel and V‐Track (Campbell et al., 2012) can avoid any human error in their calculation. However, both Daniels et al. (2019) and Notte et al. (2021) found the amount of time tags were in a predator's gut (tag retention) to be a limiting factor. In striped bass the average tag retention was 2.9 days, at which point tags were ejected from the gastrointestinal tract of the predator. Before studies were able to use predator tags, the absence of a tag in a locality meant that behavior could not be monitored long enough to identify it as a predator (Daniels et al., 2018; Klinard et al., 2019). In this study, tag retention of predator tags was found to be between 0.7 and 45 days. Size of predator can be an impacting factor on tag retention time along with several other factors: water temperature, prey size, and tag size (Halfyard et al., 2017; Schultz et al., 2015). What is certain is that each study will have a unique set of circumstances, and variables will need to be chosen carefully. In general, the use of predator tags and these types of machine learning algorithms was successful and where possible should be employed in the quality control process of detection data.

### 4.3 Salmon migration success

The methods in which telemetry studies address mortality or survival need to be carefully considered, especially as movement ecology is increasingly being used to inform management decisions (Klinard & Matley, 2020). Detection data rely on the assumption that detection of a tag is from the individual to which the tag was affixed originally. Misinterpretation of the data can therefore be easily confounded if predation is not considered (Gibson et al., 2015). Unexpected movements of salmon have been classified as predation in other studies (Kennedy et al., 2023; Lilly et al., 2022), but not all attempt to quantify this predation into survival estimates (Chaput et al., 2019). To address this, a comparison was made between a predator‐biased group and an unbiased group in which those identified as predated upon in machine learning were removed from migration success estimates. These results clearly show that by not accounting for predation, migration success can be overestimated. At a fine temporal and spatial resolution, leaving in these detections can also skew timing, residency, and habitat usage estimates (Daniels et al., 2019; Gibson et al., 2015).

Despite demonstrating that mammalian predation was more likely in the ranched cohort within the lake and estuary, losses occurred in both wild and ranched cohorts, suggesting that wild salmon may have been more susceptible to other predators. Salmon smolts migrating to the open ocean encounter various environmental conditions and predators. In a review by Thorstad et al. (2012), the highest natural smolt mortality is reported as occurring in the estuarine and marine areas close to river mouths, and predation is the main cause of this mortality; this would certainly coincide with the type of habitat that Burrishoole smolts must successfully migrate through. Interestingly, detection rates at Clare Island (marine array) were similar between wild and ranched smolts in both 2021 and 2022. This concurs with previous studies in Clew Bay where survival rates to Clare Island were found to be similar in wild and ranched salmon (Doogan et al., 2023). Thorstad et al. (2007) showed no difference in survival between hatchery‐reared and wild smolt migration from river mouth. However, other studies (Jonsson et al., 2003; Larocque et al., 2020) have found a higher survival of wild cohorts. What is clear is that both cohorts of salmon smolts face difficult hazards on their migration out of the Burrishoole Catchment, with a high percentage not reaching the open ocean. This agrees with other studies that have emphasized the high risk of mortality of salmon smolts migrating through inshore coastal waters (Crozier et al., 2018; Flávio et al., 2020; Halfyard et al., 2013; Thorstad et al., 2012).

## 5 CONCLUSION

We conclude that the use of predator tags is an essential tool in the overall validation of detection data, especially in vulnerable species such as Atlantic salmon. Their use enables a study to adopt descriptive variables, which will make the results of machine learning more robust. It validates detection data and refines survival estimates. It is important to note that each study area is different in geographical location, environmental factors, and number and type of predators. Also, the issue of the possibility that some mammalian predators can detect the tags and therefore find smolts more easily needs to be further investigated. Despite this, the use of predator tags should be considered as part of an emerging acoustic telemetry platform with potential to inform management strategies in various localities and jurisdictions.

## AUTHOR CONTRIBUTIONS

Niall Ó'Maoiléidigh, Deirdre Cotter, Ross O'Neill, and Catherine Waters designed the tagging and receiver programme; Catherine Waters, Deirdre Cotter, Alan Drumm, Joseph Cooney, Nigel Bond, and Ger Rogan carried out the fieldwork. Catherine Waters analysed the data and wrote the draft of the manuscript, with critical review and feedback from all authors.

## FUNDING INFORMATION

This study was funded by EU award IVA5060 from the Interreg 5A programme.

## Supporting information


**Figure S1a.** Predator tag 4186, all detection times within the lake and estuary 2022. Civil twilight ends at 8:57 p.m. and begins at 4:14 a.m. in this time period.
**Figure S1b.** Predator tag 3683, all detection times in the lake and estuary 2022. Civil twilight ends at 8:57 p.m. and begins at 4:14 a.m. in this time period.
**Figure S1c.** Tracks created by a triggered predator tag using Refined Shortest Path analysis. The tag was detected for 25 days on lake and estuarine receivers.
**Figure S2a.** Two predator‐tag detection plots displaying overlapping detections (2022). Lake receivers (A1), estuary receivers (A2), Inner Bay receivers (A3), and Clare Island receivers (A4).
**Figure S2b.** Three predator‐tag detection plots displaying overlapping detections (2022).
**Figure S2c.** Two V8‐tag detection plots displaying likely predation and overlapping detections after predation by the same individual (2022).
