# Supplementary material for: The use of predator tags to explain reversal movement patterns in Atlantic salmon smolts ( Salmo salar L.)
Source: J Fish Biol. 2024 Jan 16;106(5):1316–33. doi: 10.1111/jfb.15658 (PMC12120339; doi:10.1111/jfb.15658)
Supplement: Supplementary file 1 — Figure S1a. Predator tag 4186, all detection times within the lake and estuary 2022. Civil twilight ends at 8:57 p.m. and begins at 4:14 a.m. in this time period. Figure S1b. Predator tag 3683, all detection times in the lake and estuary 2022. Civil twilight ends at 8:57 p.m. and begins at 4:14 a.m. in this time period. Figure S1c. Tracks created by a triggered predator tag using Refined Shortest Path analysis. The tag was detected for 25 days on lake and estuarine receivers. Figure S2a. Two predator‐tag detection plots displaying overlapping detections (2022). Lake receivers (A1), estuary receivers (A2), Inner Bay receivers (A3), and Clare Island receivers (A4). Figure S2b. Three predator‐tag detection plots displaying overlapping detections (2022). Figure S2c. Two V8‐tag detection plots displaying likely predation and overlapping detections after predation by the same individual (2022). [file JFB-106-1316-s001.docx]

Supplementary Figures


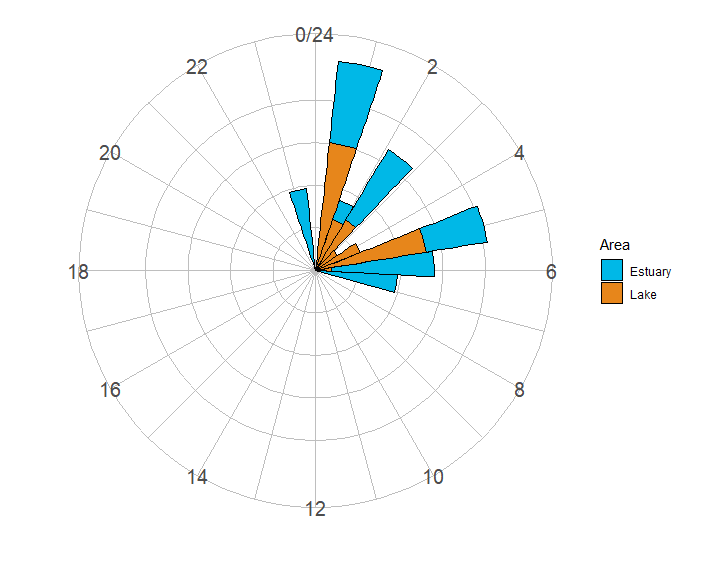


1a: Predator tag 4186, all detection times within the lake and estuary 2022. Civil twilight ends at 20:57 and begins at 04:14 in this time period.


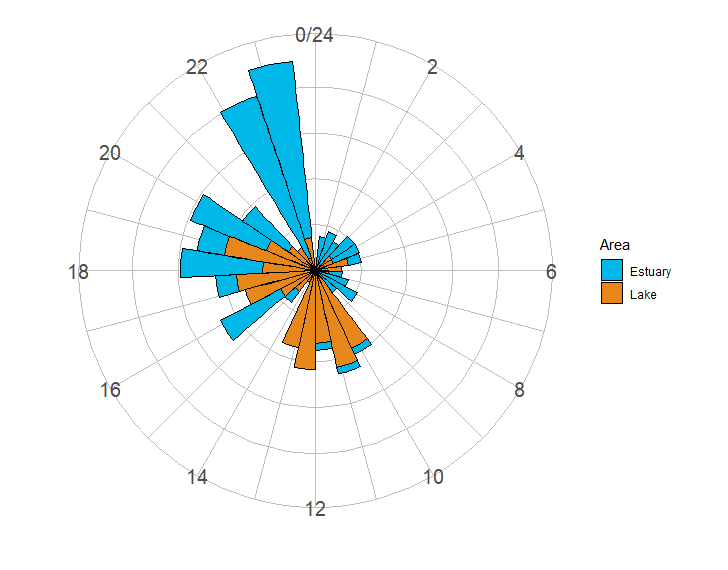
1b: Predator tag 3683, all detection times in the lake and estuary 2022. Civil twilight ends at 20:57 and begins at 04:14 in this time period.

1c: Tracks created by a triggered predator tag using Refined Shortest Path analysis. The tag was detected for 25 days on lake and estuarine receivers.


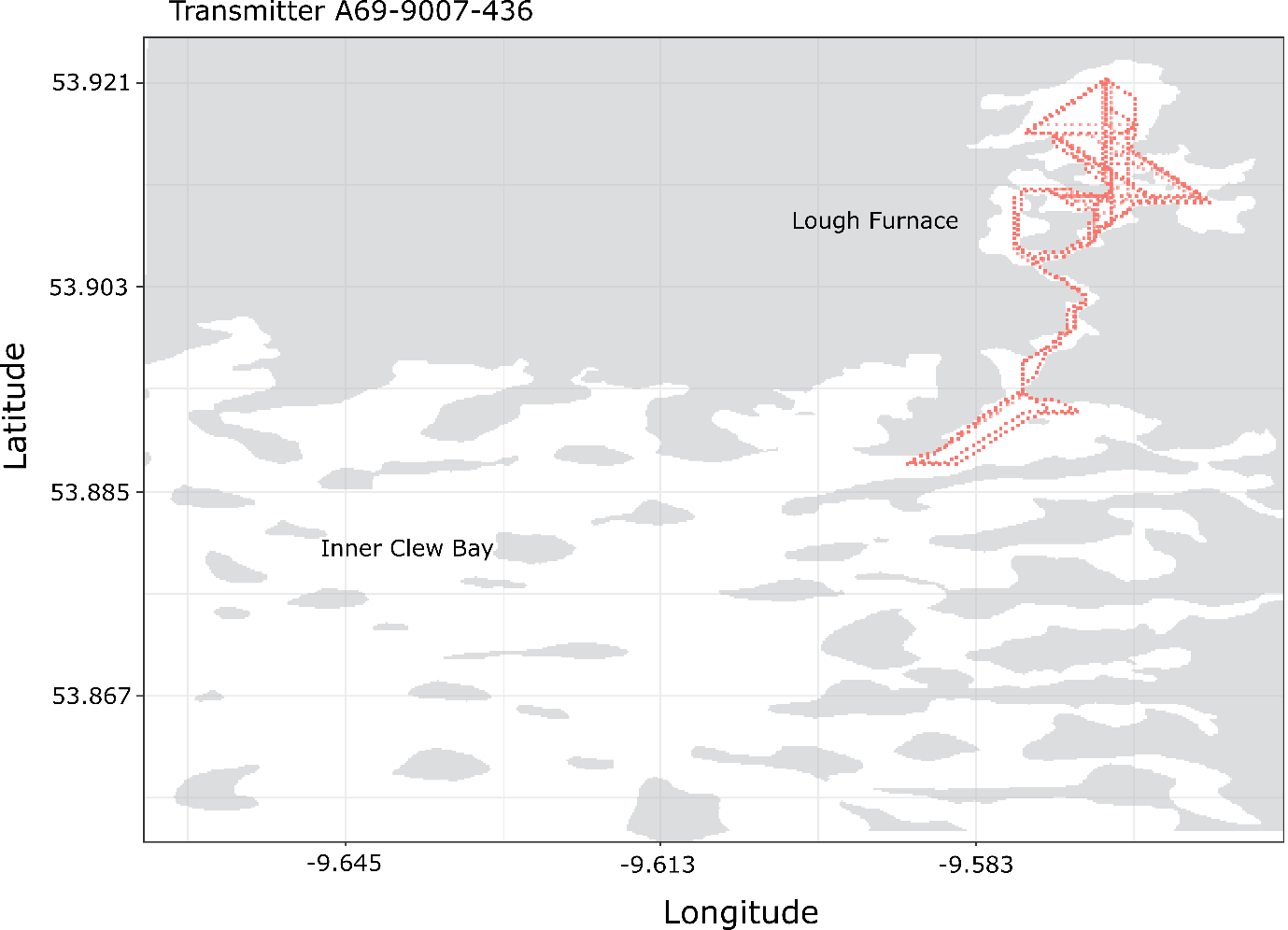


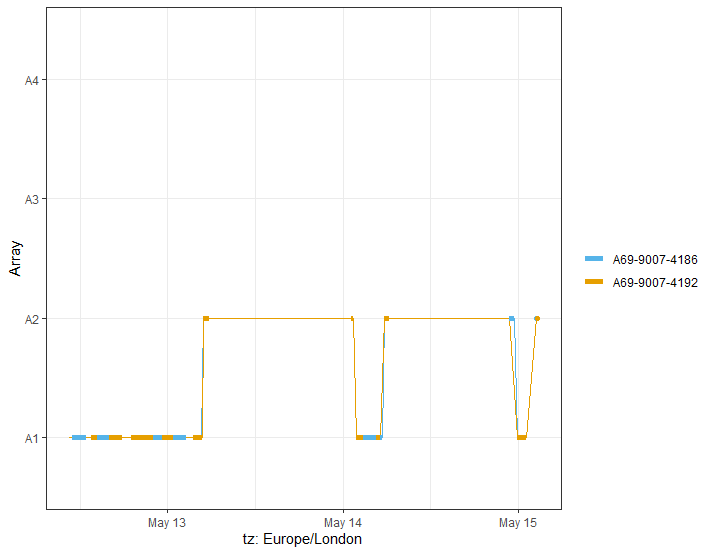


2a: Two predator-tag detection plots displaying overlapping detections (2022). Lake receivers (A1), Estuary receivers (A2), Inner Bay receivers (A3) and Clare Island receivers (A4).


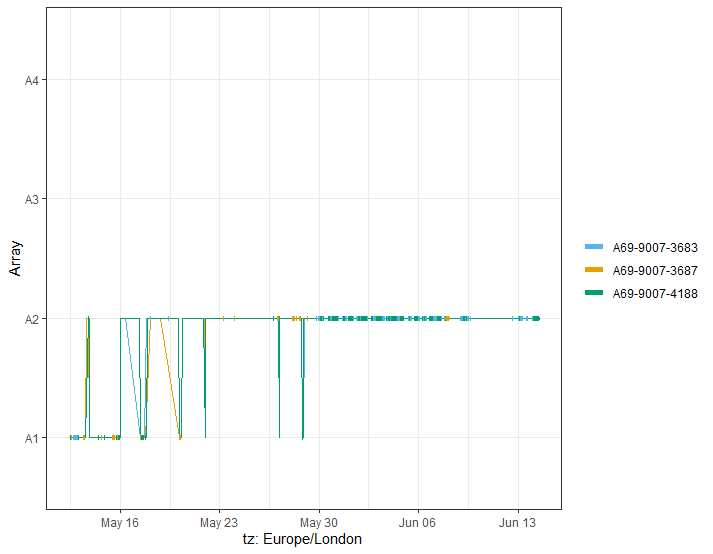


2b: Three predator-tags detection plots displaying overlapping detections (2022).


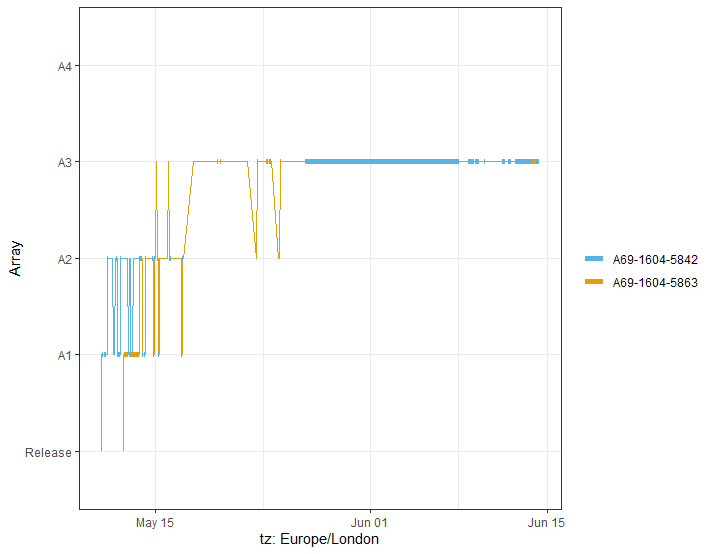


2c: Two V8-tags detection plots displaying likely predation and overlapping detections after predation by the same individual (2022).
